# Caring Under Constraints: A Qualitative Study of Parental Needs in Pediatric Diabetes in Zabol

**DOI:** 10.1155/pedi/4306754

**Published:** 2025-10-26

**Authors:** SeyedPouria Hedayati, Alireza Sotoudeh, Fatemeh Mohabati

**Affiliations:** Department of Healthcare Services Management, School of Health, Zabol University of Medical Sciences, Zabol, Iran

**Keywords:** parental needs, pediatric diabetes, qualitative study, rural health, Zabol

## Abstract

**Aim:**

This study aimed to explore the lived experiences and support needs of parents caring for children with type 1 diabetes mellitus (T1DM) in Zabol, Iran, a rural and resource-scarce region.

**Methods:**

A qualitative phenomenological design was employed with 36 parents (25 mothers and 11 fathers) of children aged 4–17 years who had lived with T1DM for at least 6 months. Semistructured interviews were conducted in Persian, transcribed, translated, and thematically analyzed using Braun and Clarke's six-step framework, in accordance with Consolidated Criteria for Reporting Qualitative Research (COREQ) reporting guidelines.

**Results:**

Three overarching themes emerged. Parents reported emotional and practical support gaps, including caregiver exhaustion, lack of respite opportunities, and limited guidance. They described social isolation and stigma driven by cultural misconceptions such as viewing diabetes as a curse, which led to exclusion of both parents and children. Families also faced healthcare system challenges, including limited specialist access, insufficient diabetes education, financial strain (2–15 million IRR monthly), and inadequate resources, all exacerbated by rural isolation.

**Conclusions:**

Parents of children with T1DM in Zabol experience substantial unmet emotional, social, and systemic needs. Addressing these challenges requires structured peer support, culturally sensitive community education to reduce stigma, and expanded access to affordable healthcare. These findings provide a foundation for developing targeted interventions to strengthen resilience and improve outcomes among families in underserved rural regions.

## 1. Introduction

The management of pediatric type 1 diabetes mellitus (T1DM) is intensive and requires continuous parental involvement, especially for younger children who depend on their caregivers for daily disease management. Parents are responsible for multiple demanding tasks, including insulin administration, blood glucose monitoring, dietary planning, and providing emotional support [1, 2]. These responsibilities extend beyond medical routines and often necessitate balancing clinical tasks with the child's psychological and social well-being, which can be profoundly affected by the burden of diabetes care [3, 4]. Effective and consistent management is essential for maintaining optimal glycemic control and preventing both acute and long-term complications. International clinical guidelines, including those from the International Society for Pediatric and Adolescent Diabetes (ISPAD) and the American Diabetes Association (ADA), emphasize the importance of individualized insulin therapy, structured diabetes education, and careful monitoring to support both medical outcomes and psychosocial adjustment [5, 6].

Effective management strategies for pediatric T1DM include individualized insulin therapy, medical nutrition therapy, and promotion of regular physical activity, as recommended by international guidelines [7, 8]. Collaboration between healthcare providers and caregivers is critical, as parental involvement directly influences children's health outcomes [9]. Parents frequently experience chronic sorrow, anxiety, and helplessness, which can lead to stigma and social isolation, reducing family and community support [10, 11]. In resource-scarce rural contexts, these challenges are compounded by logistical barriers such as limited healthcare access and financial strain due to the high costs of insulin and supplies [12].

Parental stress has been linked to children's glycemic control, with higher caregiver stress correlating with elevated HbA1c levels in children [13, 14]. Existing instruments, such as the Pediatric Inventory for Parents (PIP), provide a structured way to measure caregiver stress but may not fully capture the broader social dynamics that shape family resilience [15]. Complementary strategies, including peer support programs, family-centered interventions, and community education, have shown promise in improving coping capacity and mitigating stigma [16, 17]. Despite the rising global incidence of T1DM, particularly in low- and middle-income countries (LMICs), there is limited evidence on the supportive needs of parents in rural Iran. This gap highlights the importance of context-specific research to understand the lived experiences of families in resource-limited regions such as Zabol, where cultural, economic, and systemic factors significantly affect diabetes care. Therefore, this study sought to address the question: “what are the supportive and social needs of parents of children with T1DM in their daily lives in Zabol?” By qualitatively examining lived experiences, this research aims to provide insights that can inform tailored interventions to improve resilience, care access, and child outcomes in underserved rural contexts.

## 2. Methods

### 2.1. Study Design

We conducted a qualitative phenomenological study under an interpretivist paradigm, viewing parental needs as contextually shaped [18]. This approach highlights the complexities of managing pediatric diabetes and the unique experiences of parents navigating this chronic condition [19]. The study adhered to the Consolidated Criteria for Reporting Qualitative Research (COREQ) checklist to ensure transparency and rigor in reporting, which is essential for enhancing the credibility of qualitative research findings [20].

### 2.2. Setting

The study was carried out in Zabol, a city in southeast Iran near the Afghanistan border. Zabol is characterized by limited healthcare infrastructure, long distances to tertiary centers (e.g., nearest endocrinologist in Zahedan, ~2–3 h away), and a largely low- to middle-income population. These contextual features make it a relevant setting for studying parental caregiving challenges in type 1 diabetes.

### 2.3. Participants and Recruitment

Thirty-six parents (25 mothers and 11 fathers) of children with T1DM were recruited from the pediatric and general wards of Zabol hospitals between December 2024 and March 2025. Inclusion criteria were: being the primary caregiver of a child under 18 diagnosed with T1DM for ≥6 months, and ability to participate in an interview in Persian. Informed consent was obtained from all participants prior to participation, in accordance with ethical requirements, but was not used as an inclusion criterion.

Purposive sampling was applied to capture variation in parental perspectives (e.g., child age, caregiver gender, and socioeconomic background) [21]. Stratification by urban versus rural residence was not applied due to the predominantly rural and semiurban nature of Zabol's population; however, geographic context (travel distance and healthcare access) was considered during analysis [22]. Data saturation was achieved after 33 interviews, confirmed by three additional interviews, indicating that no new themes were emerging from the data [23].

### 2.4. Data Collection

Semistructured interviews lasting 45–60 min were conducted in Persian by two trained researchers fluent in the Zabol dialect. The interview guide was informed by prior studies [24, 25], pilot-tested with three local parents, and adapted with probes relevant to Zabol's context (e.g., travel costs and community beliefs). Interviews were audio-recorded, transcribed verbatim, and translated into English with dual verification for accuracy. Field notes captured nonverbal cues.

### 2.5. Data Analysis

Data were analyzed using Braun and Clarke's six-step thematic analysis framework [26]. Coding was conducted in NVivo 12 by two researchers, achieving 92% agreement on a 30% subset of transcripts. Themes were refined through iterative discussion.

### 2.6. Rigor

Credibility was established through member checking with six parents of children with T1DM, who confirmed that the identified themes accurately reflected their experiences. Dependability and confirmability were supported through detailed audit trails and peer debriefing sessions with diabetes care experts. Transferability was enhanced by providing rich descriptions of the study setting, participants' caregiving experiences, and local healthcare challenges.

## 3. Results

### 3.1. Participant Characteristics

A total of 36 parents participated in this study, including 25 mothers and 11 fathers, all serving as primary caregivers of children aged 4–17 years living with T1DM for at least 6 months. Families self-identified as low to middle income. Recruitment was conducted in pediatric and general wards of Zabol hospitals. Participant characteristics are summarized in Table 1.

### 3.2. Themes

Three core themes were identified: (1) Emotional and practical support gaps, (2) social isolation and stigma, and (3) navigating healthcare systems. Each theme is illustrated with subthemes and representative quotations. The summary of these themes (provided with subthemes below and in Table 2) describe how parental experiences are shaped by rural isolation and cultural norms encapsulated in resource constraints.

#### 3.2.1. Emotional and Practical Support Gaps

Parents described overwhelming emotional strain and insufficient support.

• Emotional overload: Caregivers, especially mothers, worried constantly about their child's future. One mother of a 7-year-old reported: “I cry all alone at night, wondering if she'll live a normal life; no one sees the weight.”
• Lack of family support: Relatives often misunderstood diabetes, offering little assistance. A father noted: “My family blames me; they think she is sick because I failed.”
• Need for respite: Many parents expressed exhaustion and desire for relief. A mother of a 12-year-old said: “I just need a day where someone else takes over.”
• Desire for guidance: Parents wanted practical advice from peers and professionals. A father of a 5-year-old stated: “I'd feel less lost if I could talk to another parent who has done this.”

#### 3.2.2. Social Isolation and Stigma

Parents reported exclusion due to misconceptions and cultural stigma.

• Community misunderstanding: Diabetes was often viewed as contagious or a parental failing. One mother shared: “They say I fed him too much sugar; now children stay away from him.”
• Child exclusion: Several children were isolated at school or play. A father of an 8-year-old explained: “Other kids won't play with him; they think he's always sick.”
• Parental withdrawal: Stigma and judgment led parents to retreat from social activities. A mother commented: “I stopped attending gatherings; no one understands what we face.”
• Cultural stigma: Diabetes was described as a “curse” within traditional beliefs. A father said: “People call it a curse, so I hide her condition from relatives.”

#### 3.2.3. Navigating Healthcare Systems

Parents highlighted structural barriers to accessing diabetes care.

• Limited access to specialists: The nearest pediatric endocrinologist was located in Zahedan, about 3 h away. A mother explained: *“*The nearest specialist is in Zahedan; we can't afford the trip.”
• Inadequate education: Families felt unprepared to manage T1DM after diagnosis. A father recalled: “They handed me a pamphlet and sent me home; I didn't know where to start.”
• Resource scarcity: Essential supplies, such as glucose test strips, were often unavailable. A mother of a 14-year-old said: “When the pharmacy runs out of strips, I panic not knowing his levels.”
• Financial strain: Monthly costs of 2–15 million IRR placed severe burdens on families. A parent of two diabetic children shared: “We spend 10 million on insulin and sensors—it's crushing. Sometimes we skip meals to afford her medicine.”

## 4. Discussion

This study of 36 parents of children with T1DM in Zabol, Iran, highlights the multifaceted challenges families face in emotional, social, and systemic domains. The three themes, emotional and practical support gaps, social isolation and stigma, and navigating healthcare systems, demonstrate how the rural and resource-limited context intensifies parental burdens.

### 4.1. Emotional and Practical Strain

The findings on emotional and practical strain among caregivers in Zabol are consistent with evidence from other resource-constrained settings, where parents frequently report exhaustion and limited respite. For example, caregivers of children with cerebral palsy in Zimbabwe reported similar distress and an unmet need for support [27]. Such challenges reflect a broader pattern across low-resource contexts where inadequate systems intensify caregiver burden. However, unlike many urban areas, Zabol lacks structured peer networks and formal support groups. In urban India, mobile health applications and community-based programs have been shown to ease caregiver strain and improve access to information [28].

This absence of support structures in Zabol exacerbates caregivers' isolation and heightens risks of poor mental health, a link well established in the literature [29]. The findings therefore emphasize the urgent need for targeted interventions that reflect the specific realities of rural and underserved communities. Community-based initiatives and outreach services can play a vital role in improving caregiver well-being and resilience [30]. Addressing these gaps could help mitigate the unique challenges faced by families in Zabol while aligning their experiences with broader global insights.

### 4.2. Social Isolation and Stigma

In Zabol, families of children with T1DM face profound social isolation and stigma driven by cultural misconceptions that portray diabetes as a “curse” or parental failing. This cultural framing differs significantly from Western literature, where stigma around diabetes typically involves public misunderstanding or discrimination rather than spiritual blame [30]. Studies from India and the Middle East reveal similar stigma-related challenges, yet Zabol's findings highlight more severe consequences, such as children's exclusion from school and parents' withdrawal from community life. For example, research in India links diabetes stigma to poorer glycemic control but rarely emphasizes such extreme social isolation [31].

The findings emphasize the need for culturally tailored interventions to address stigma's unique manifestations in Zabol. Unlike some regions where public disclosure and advocacy reduce stigma [32], shame and secrecy in Zabol deter families from seeking support, mirroring patterns observed in studies of mental illness stigma [33]. Effective interventions could include community education workshops to dispel diabetes misconceptions, peer support groups for parents, and school-based programs to prevent discrimination against affected children [34].

### 4.3. Healthcare Navigation Challenges

The challenges of navigating healthcare in Zabol, particularly for diabetes management, align with global patterns observed in rural healthcare systems. Limited access to specialists, inadequate initial education, and unreliable availability of medical supplies are recurring issues across LMICs. A cross-sectional study in 42 LMICs demonstrated that rural populations are significantly less likely to meet critical diabetes management benchmarks compared to urban populations [35].

The economic burden in Zabol, with monthly costs ranging from 2 to 15 million IRR, highlights a particularly severe financial strain compared to other rural settings. While economic barriers are common, the acute financial distress in Zabol often forces families into extreme measures such as forgoing meals, which is less frequently reported elsewhere. For instance, a qualitative study from Nepal identified financial barriers to diabetes care, but did not describe such extreme consequences [36]. Furthermore, Zabol's cultural and systemic factors create a unique caregiving landscape. While parental distress is a common challenge in rural diabetes care, Zabol's cultural context may intensify these issues. Research from rural Australia shows how health literacy and socioeconomic factors interact, suggesting that leveraging community strengths could enhance healthcare access [37].

In conclusion, the healthcare navigation challenges in Zabol reflect global trends in rural diabetes care, but are exacerbated by unique economic and cultural factors. Understanding these nuances is critical for designing targeted strategies to improve diabetes management in similar rural contexts.

### 4.4. Implications for Practice and Policy

Based on our findings, several targeted strategies are recommended:

1. Telehealth and mobile clinics: Implementing telehealth services and mobile clinics can significantly reduce geographic barriers and expand access to pediatric endocrinologists. Evidence demonstrates that telehealth improves appointment adherence, patient satisfaction, and quality of life in youth with diabetes [38, 39].
2. Community education programs: Establishing education initiatives led by trusted local leaders can help dispel myths, reduce stigma, and foster inclusion of children with diabetes. Studies show that integrating family and community support with education enhances engagement and self-management [40, 41].
3. Psychosocial support interventions: Counseling and peer support groups are crucial for addressing caregiver burnout and emotional distress. Research indicates that psychosocial screening and interventions improve quality of life for both caregivers and children [42, 43].
4. Financial and supply chain support: Providing subsidies for insulin and test strips, alongside reliable supply chain management, is essential. Evidence highlights the importance of financial risk protection strategies to reduce economic burden [44].
5. Family-based education: Structured family education programs can improve knowledge, coping, and shared responsibility. Evidence shows such programs enhance glycemic control and self-efficacy when tailored to cultural needs [45, 46].

Together, these strategies aim to improve diabetes care in rural settings like Zabol by addressing systemic barriers and the unique cultural context.

## 5. Conclusion

Parents of children with T1DM in Zabol may face difficulties in integrating their families into the community due to cultural stigma and rural isolation. The role of healthcare providers and community educators in facilitating families' adjustment is significant. Providers may offer diabetes education to parents and extended family members, while also helping children and caregivers accept the condition and manage it more efficiently. Parents' general need for empathic support means that they seek attention to emotional well-being, flexibility in care routines, communication with specialists about the child's condition, and assistance with tasks around blood glucose control and insulin administration, often with help from peer networks or telehealth. The findings of the present study may guide more detailed examinations of associations among psychological, motivational, and environmental factors in the subject of diabetes management in rural, resource-limited settings. Training programs for caregivers and community members on diabetes management, led by professionals, may benefit from our study. Based on our results, more emphasis should be placed on issues that cause distress and burden for parents (e.g., what to do in case of acute hypoglycemia or hyperglycemia, managing financial strain from monthly costs of 2–15 million IRR) and the role of parents in providing emotional support to their children with diabetes (e.g., acceptance of T1DM, stigma reduction through community sensitization, and peer education).

## 6. Limitations and Future Directions

This study is limited by its focus on one rural region in Iran, which may reduce transferability to other contexts. The sample size, while adequate for thematic saturation, reflects only parents and not children or healthcare providers. Future research should expand to multiple regions, include diverse stakeholder perspectives, and evaluate the effectiveness of proposed interventions in practice.

## Figures and Tables

**Table 1:** Participant characteristics (*n* = 36).

Characteristic	Details
Number of participants	36
Parent gender	25 Mothers and 11 fathers
Child age range	4–17 years
Duration of child's T1DM	≥6 months
Socioeconomic status	Low to middle income (self-reported)
Recruitment site	Zabol hospitals (pediatric and general wards)
Caregiver role	All primary caregivers

*Note*: Gender and age: from methods: “25 mothers and 11 fathers; child age 4–17 years.” Socioeconomic status: from methods: “low to middle income, assessed via self-report.” Excluded nonessential details (e.g., exact income) to keep it concise for journal readers.

**Table 2:** A summary of the three major themes, subthemes, and illustrative quotations.

Theme	Subtheme	Description	Representative quote
Emotional and practical support gaps	Emotional overload	Parents overwhelmed by fear and stress related to diabetes care	“I cry alone, wondering if she'll live a normal life.” (Mother of a 7-year-old)
	Lack of family support	Limited assistance due to relatives' misunderstanding	“My family thinks it's my fault.” (Father)
	Need for respite	Exhaustion and desire for caregiving relief	“I just need a day where someone else takes over.” (Mother of a 12-year-old)
	Desire for guidance	Seeking advice from peers or professionals	“I'd feel less lost if I could talk to another parent.” (Father of a 5-year-old)

Social isolation and stigma	Community misunderstanding	Misconceptions leading to family isolation	“They say I fed him too much sugar.” (Mother)
	Child's exclusion	Rejection at school or among peers	“Other kids won't play with him.” (Father of an 8-year-old)
	Parental withdrawal	Parents avoiding social gatherings due to judgment	“I don't go to gatherings anymore.” (Mother)
	Cultural stigma	Diabetes viewed as shameful or a curse	“People call it a curse.” (Father)

Healthcare system challenges	Limited access to specialists	Long travel distances hinder access to care	“The nearest specialist is three hours away.” (Mother)
	Inadequate education	Insufficient training provided at diagnosis	“They handed me a pamphlet.” (Father)
	Resource scarcity	Frequent shortages of supplies	“The pharmacy runs out of strips—I panic.” (Mother of a 14-year-old)
	Financial strain	High monthly costs of insulin and supplies	“We spend 10 million rials on insulin and sensors.” (Parent, interview 5)

## Data Availability

The data that support the findings of this study are available from the corresponding author upon reasonable request.
